# Prayer Made Her Whole

**Published:** 1892-01

**Authors:** 


### PRAYER MADE HER WHOLE.

A sensation occurred in New Brunswick, N. J., last July, by the miraculous cure of Mrs. Mary Paul, who has been bedridden and a victim of cancer for ten years. Within three years Mrs. Paul had two cancers removed from her breast, and her death was looked upon as likely to occur at any moment.

While seated with her son-in-law, Captain Johnson, at the breakfast table a few days ago, Mrs. Paul suddenly cried out: "Oh, God, heal my poor weak body." A moment later after a silent prayer she again cried: "Thank God, I am healed." Previous to that moment Mrs Paul had only been able to go from her bed to the table. She had not performed any household duties in many years.

Mrs. Johnson, her daughter, related the story of her cure. She said her mother arose from the table, threw her medicine out of the window and is now well and strong. Her face has a healthy color and she does hard work.

Mrs. Paul is 56. She came to New Brunswick from Detroit and was known for her piety. She walked to the Salvation Army barracks and told the story of her wonderful cure and left for Cedar Dale, Ontario, Canada, where she says she will tell the people of the faith that made her body whole. Mr. Johnson's neighbors fully corroborate the story of Mrs. Paul's long and apparently hopeless illness, of her inability to do any kind of work and of her sudden and miraculous cure.

